# Supplementary material for: G12/13-mediated signaling stimulates hepatic glucose production and has a major impact on whole body glucose homeostasis
Source: Nat Commun. 2024 Nov 19;15:9996. doi: 10.1038/s41467-024-54299-7 (PMC11574106; doi:10.1038/s41467-024-54299-7)
Supplement: Supplementary file 1 — Supplementary Information [file 41467_2024_54299_MOESM1_ESM.pdf]

Supplemental Information

$G_{12/13}$-mediated signaling stimulates hepatic glucose production and has a major impact on whole body glucose homeostasis

Srinivas Pittala[1], Dhanush Haspula[1], Yinghong Cui[1], Won-Mo Yang[2], Young-Bum Kim[2], Roger J. Davis[3], Allison Wing[4], Yaron Rotman[4], Owen P. McGuinness[5], Asuka Inoue[6], and Jürgen Wess[1]

[1]Molucelar Signaling Section, Laboratory of Bioorganic Chemistry, NIDDK, NIH, Bethesda, MD, USA

[2]Division of Endocrinology, Diabetes, and Metabolism, Beth Israel Deaconess Medical Center and Harvard Medical School, Boston, MA, USA

[3]Program in Molecular Medicine, University of Massachusetts Chan Medical School, Worcester, MA, USA

[4]Liver & Energy Metabolism Section, Liver Diseases Branch, NIDDK, NIH, Bethesda, MD, USA

[5]Departments of Molecular Physiology and Biophysics, Vanderbilt University Medical Center, Nashville, TN, USA

[5]Graduate School of Pharmaceutical Sciences, Tohoku University, Sendai, Miyagi, 980-8578, Japan

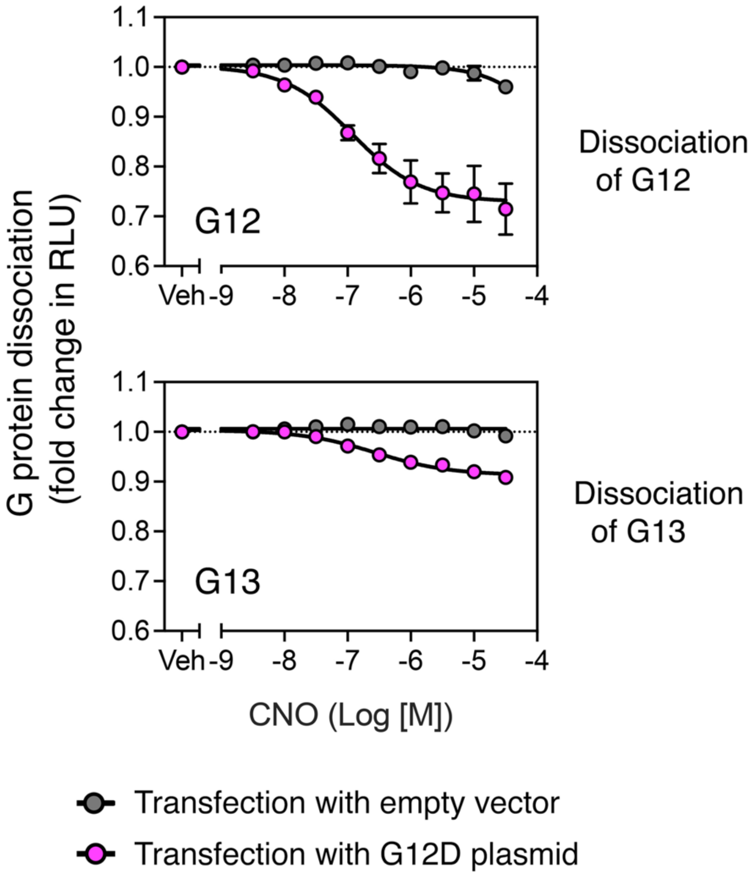

Supplementary Fig. 1. **CNO treatment of G12D-expressing HEK293A cells activates both G_{12} and G_{13}.** CNO-induced activation of G_{12} and G_{13} in HEK293A cells expressing the G12D designer GPCR[1]. G12D-mediated dissociation of G protein heterotrimers was measured by employing a highly sensitive NanoBiT-G protein dissociation assay (see Methods for details)[2]. Data are presented as means ± s.e.m. obtained in 3-5 independent experiments. Veh, vehicle; RLU, relative luminescence units. Source data are provided as a Source Data file.

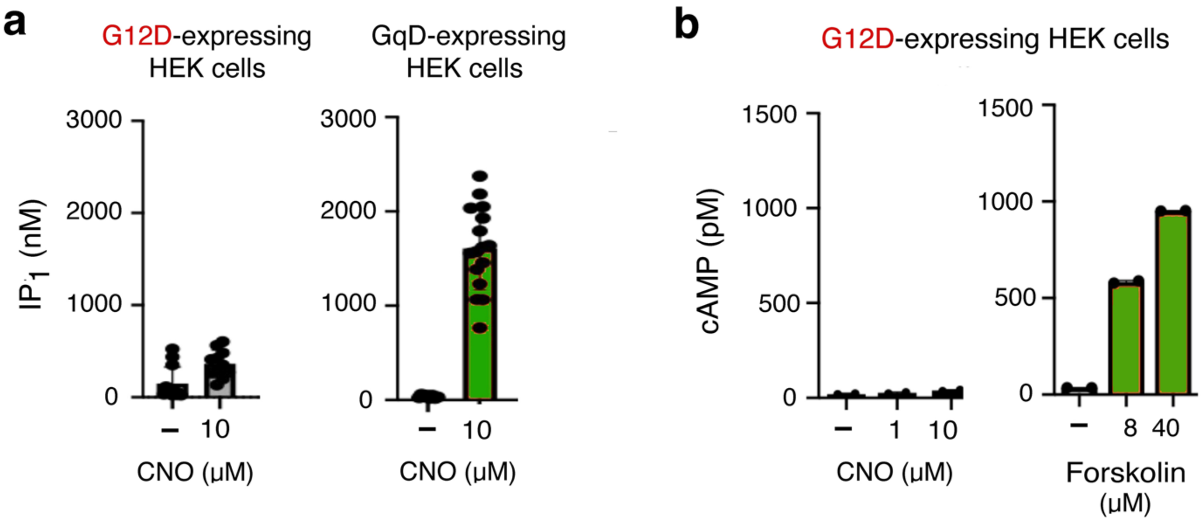

Supplementary Fig. 2. CNO treatment of G12D-expressing HEK293A cells does not lead to increased intracellular inositol monophosphate (IP_1) and cAMP levels. **a, b,** G12D-expressing HEK293A cells were incubated with the indicated concentrations of CNO or forskolin for 30 min, and changes in cytosolic IP_1 (**a**) and cAMP (**b**) levels were determined (see Methods for details). For control purposes, we also studied HEK293A cells expressing a $G_{q/11}$-coupled DREADD (hM3Dq; alternative name: GqD)[3] (**a**). Data are presented as means ± s.e.m. obtained in three independent experiments. Source data are provided as a Source Data file.

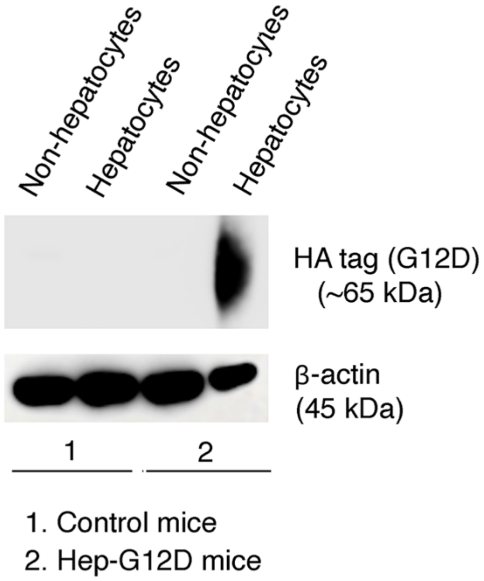

Supplementary Fig. 3. **Selective expression of the G12D designer receptor in hepatocytes of Hep-G12D mice.** Hepatocytes were prepared from Hep-G12D mice and control littermates and separated from other liver cells (Kupffer cells, stellate cells, etc.). Western blotting studies with cell lysates showed that G12D was selectively expressed in hepatocytes of Hep-G12D mice. The G12D protein was detected by using an anti-HA antibody targeting the HA epitope fused to the extracellular N-terminus of G12D (Fig. 1a). A representative blot is shown. Two additional experiments gave similar results. Source data are provided as a Source Data file.

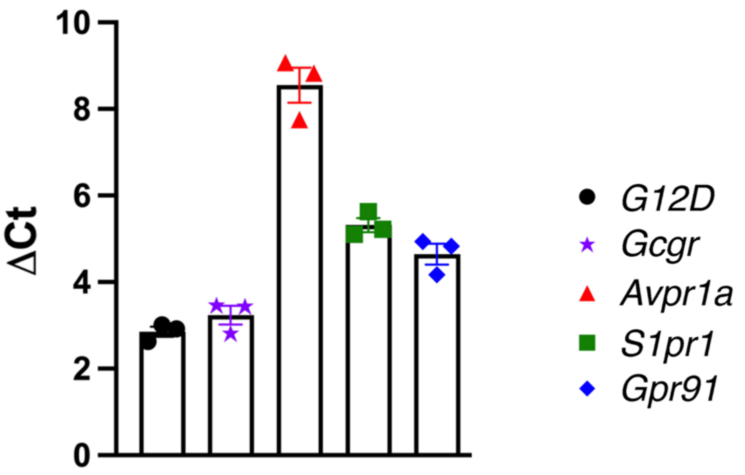

Supplementary Fig. 4. Quantification of hepatic expression levels of G12D and several GPCRs endogenously expressed by mouse hepatocytes. RNA prepared from primary hepatocytes derived from Hep-G12D mice was subjected to qRT-PCR analysis using receptor-specific primers. We compared the expression of *G12D* with that of the following GPCRs (gene names in paratheses): glucagon receptor (*Gcgr*), V_{1A} vasopressin receptor (*Avpr1a*), sphingosine 1-phosphate receptor subtype 1 (*S1pr1*), and Gpr91 (*Sucnr1*). Expression data were normalized relative to the expression of β-actin (internal control). Note that lower ΔC_t values correspond to higher transcript levels. Data are presented as means ± s.e.m. obtained in three independent experiments. Source data are provided as a Source Data file.

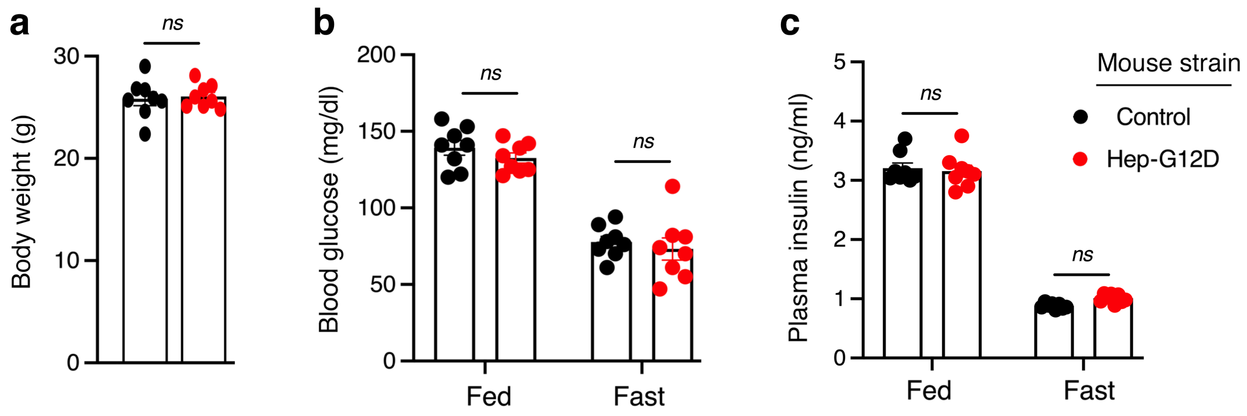

Supplementary Fig. 5. **Studies with Hep-G12D mice and control littermates in the absence of CNO.** Metabolic measurements performed with Hep-G12D mice and control littermates maintained on regular chow in the absence of CNO. **a,** Body weight (age: 8 weeks). **b, c,** Blood glucose (b) and plasma insulin (c) levels in mice that had free access to food or after a 14 hr overnight fast. All studies were performed using 8-9-week-old male mice. Data represent means ± s.e.m. (n=8/group). ns, no statistically significant difference (two-tailed Student's t test [a] or 2-way ANOVA followed by Bonferroni's post-hoc test [b, c]). Source data are provided as a Source Data file.

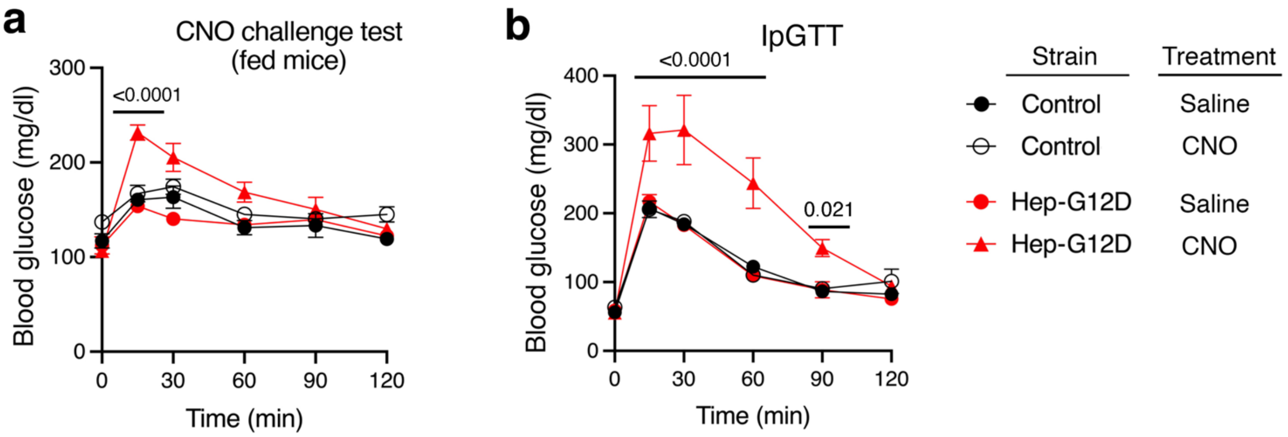

Supplementary Fig. 6. In vivo metabolic studies with female Hep-G12D mice. In vivo metabolic tests were performed with female Hep-G12D mice and control littermates (age: 3-4 months) maintained on regular chow. **a,** CNO challenge test. Freely fed mice were injected i. p. with CNO (3 mg/kg) or saline, followed by monitoring of blood glucose levels. **b,** I.p. glucose tolerance test (IpGTT, 2 g glucose/kg). Data represent means ± s.e.m. (n=8 mice/group). Numbers above horizontal bars refer to p values. Statistical significance was determined by 2-way ANOVA followed by Bonferroni's post-hoc test. Source data are provided as a Source Data file.

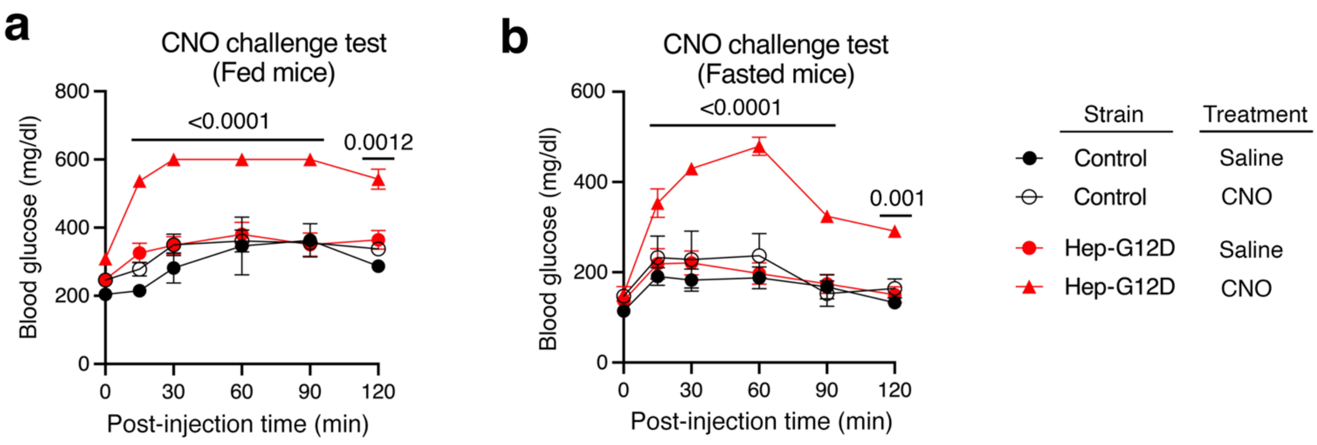

Supplementary Fig. 7. CNO treatment of Hep-G12D mice maintained on a high fat diet. **a, b,** CNO challenge tests. Hep-G12D mice and control littermates were maintained on a high fat diet for at least 8 weeks. Mice that had free access to food (**a**) or that had been fasted overnight for 12 hr (**b**) were injected i. p. with CNO (3 mg/kg) or saline, followed by the monitoring of blood glucose levels. Studies were performed using male mice (mouse age: 16-17 weeks). Data represent means ± s.e.m. (n=8 mice/group). Numbers above horizontal bars refer to p values. Statistical significance was determined 2-way ANOVA followed by Bonferroni's post-hoc test. Source data are provided as a Source Data file.

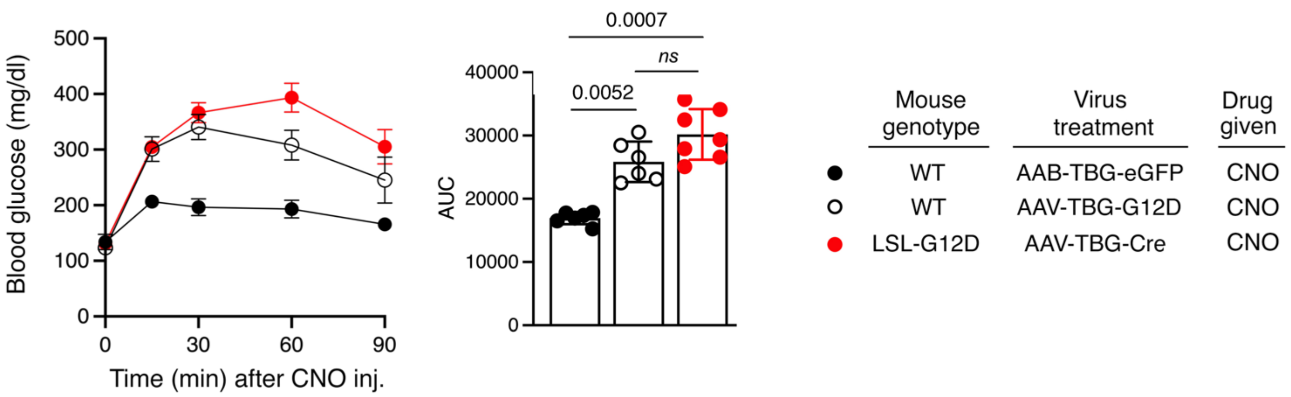

Supplementary Fig. 8. **Hyperglycemic responses after ligand activation of the G12D designer receptor expressed in mouse hepatocytes.** The G12D construct was expressed selectively in mouse hepatocytes by using two related strategies. One approach involved the treatment of LSL-G12D mice with the AAV-TBG-Cre virus. The second strategy involved the treatment of WT mice with the AAV- TBG-G12D virus (see Methods for details). The resulting mice showed comparable increases in blood glucose levels following the i.p. jection of CNO (3 mg/kg). Studies were performed using 8-week-old male mice consuming regular chow diet. Data are given means ± s.e.m. (n=8 mice/group). Numbers above horizontal bars refer to p values (2-way ANOVA followed by Bonferroni's post-hoc test). ns, no statistically significant difference. Source data are provided as a Source Data file.

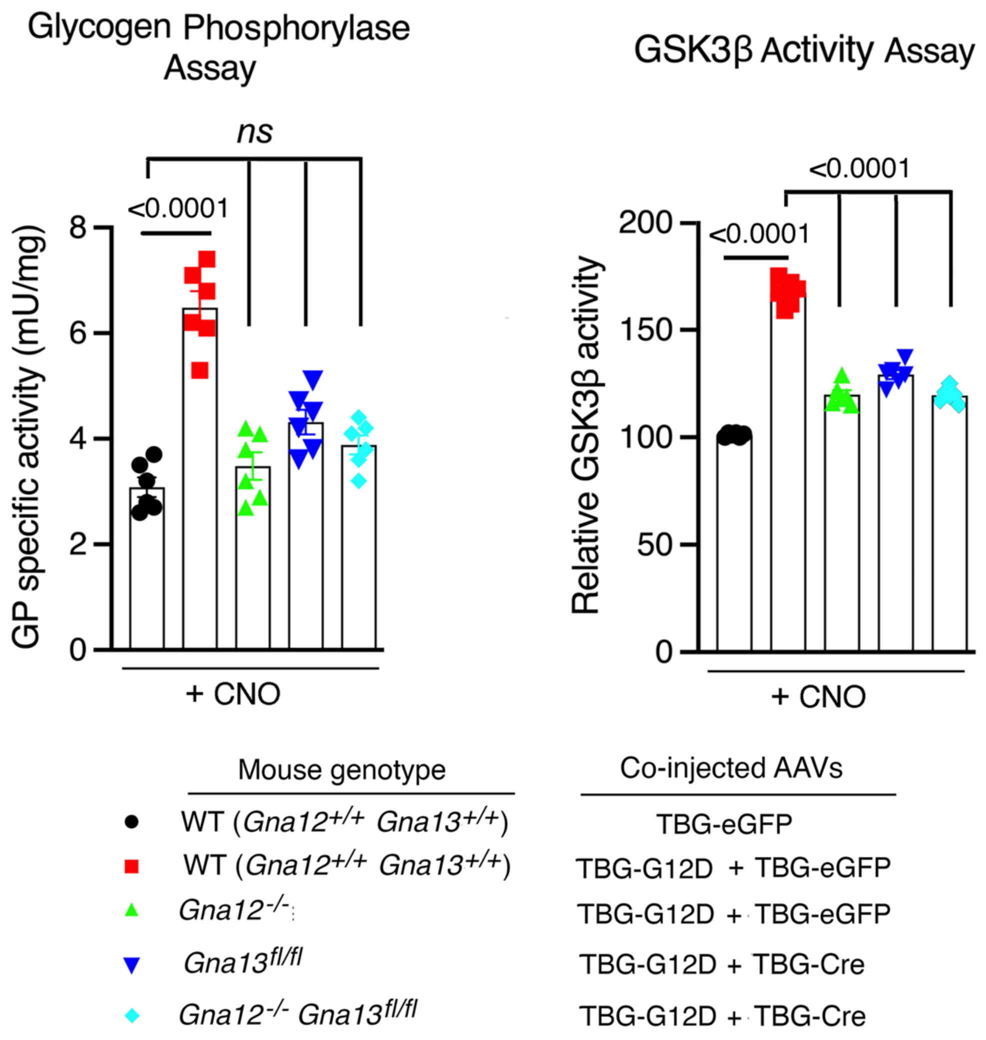

Supplementary Fig. 9. **Both G_{12} and G_{13} signaling contribute to G12D-mediated stimulation of hepatic glycogenolysis.** Livers were harvested from the indicated mouse strains 30 min after CNO treatment (3 mg/kg, i.p.) 14-week-old males). The activities of hepatic glycogen phosphorylase (GP) and Gsk3β, two enzymes that play central roles in promoting glycogenolysis, were determined using liver lysates. Data are given means ± s.e.m. (n=6). Numbers above horizontal bars refer to p values (2-way ANOVA followed by Bonferroni's post-hoc test). Source data are provided as a Source Data file.

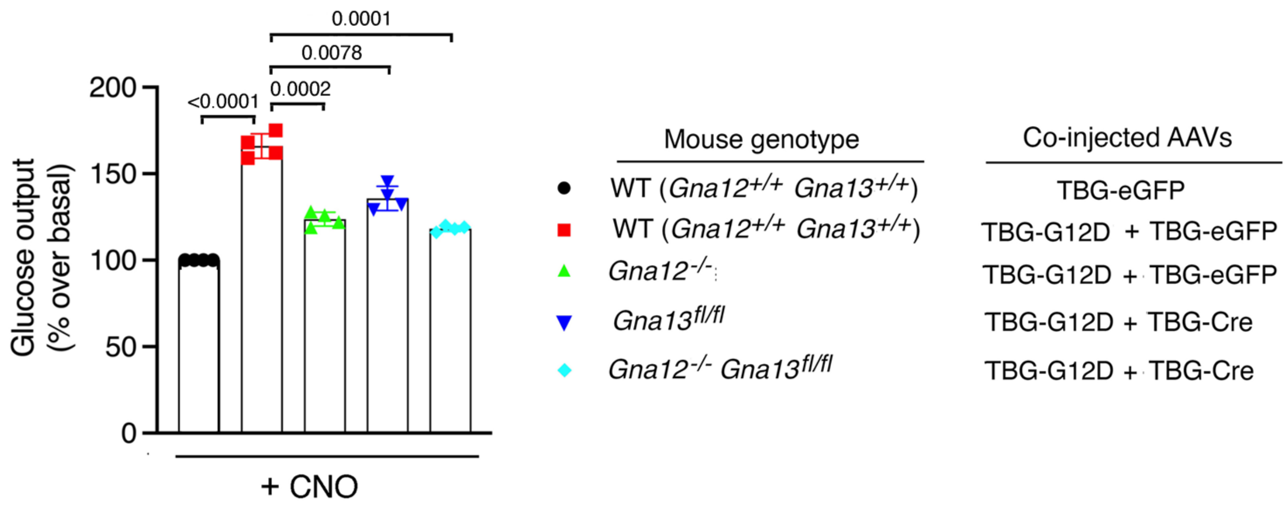

Supplementary Fig. 10. **Both G_{12} and G_{13} signaling contribute to G12D-mediated stimulation of hepatic gluconeogenesis.** We collected hepatocytes from livers of the indicated mouse strains 30 min after CNO treatment (3 mg/kg, i.p.) (14-week-old males). To monitor gluconeogenesis, we incubated hepatocytes in the presence of CNO (10 µM) and high concentrations of two major gluconeogenic substrates (20 mM sodium lactate and 2 mM sodium pyruvate, respectively) and measured the amount of glucose released into the medium. Data are given means ± s.e.m. (n=4). Numbers above horizontal bars refer to p values (2-way ANOVA followed by Bonferroni's post-hoc test). Source data are provided as a Source Data file.

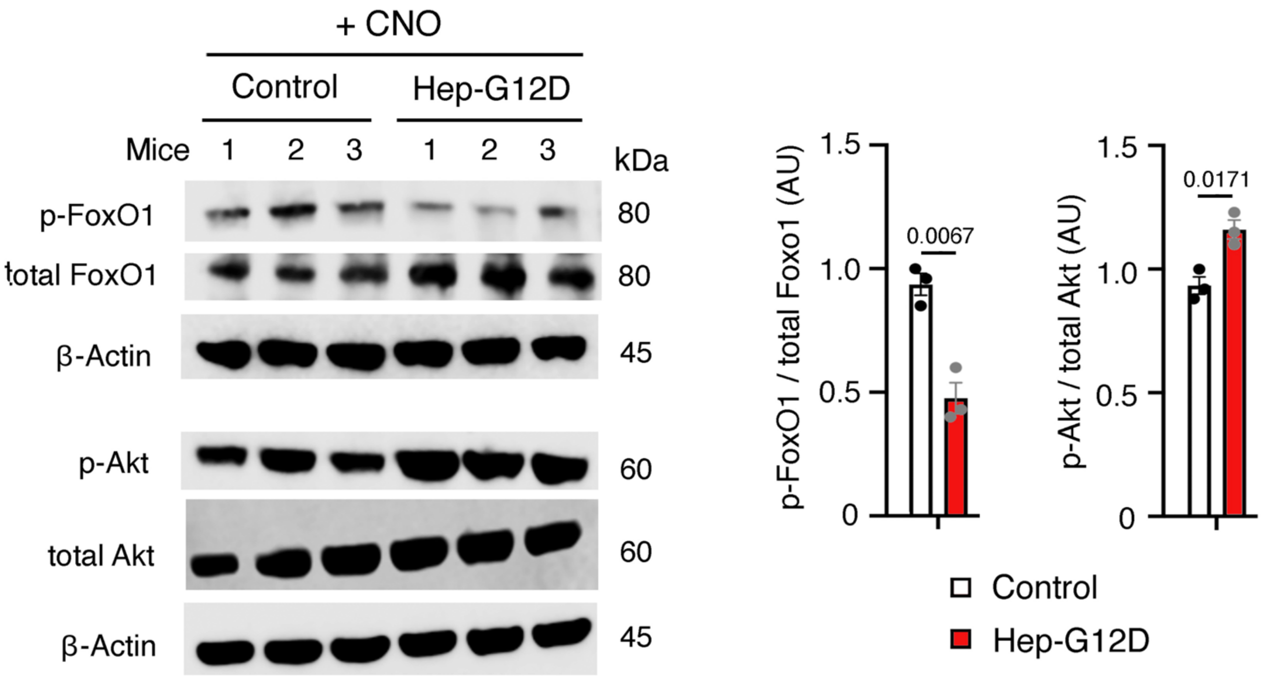

Supplementary Fig. 11. Activation of G12D expressed by mouse hepatocytes promotes the dephosphorylation of pFoxO1. HepG12D mice and control littermates (16-week-old males) were injected with CNO (3 mg/kg, i.p.). Thirty min later, mice were euthanized, and liver lysates were prepared and subjected to Western blotting studies using the indicated antibodies. Note that CNO treatment of Hep-G12D mice caused a marked reduction in hepatic pFoxO1 levels. pAkt (T308) was slightly elevated in Hep-G12D mice, most likely due to enhanced insulin secretion following CNO-induced hyperglycemia (see text for details). Western blotting data are quantified in the bar diagrams to the right (n=3 mice per group). Data represent means ± s.e.m. Numbers above horizontal bars refer to p values (two-tailed Student's t test). Source data are provided as a Source Data file.

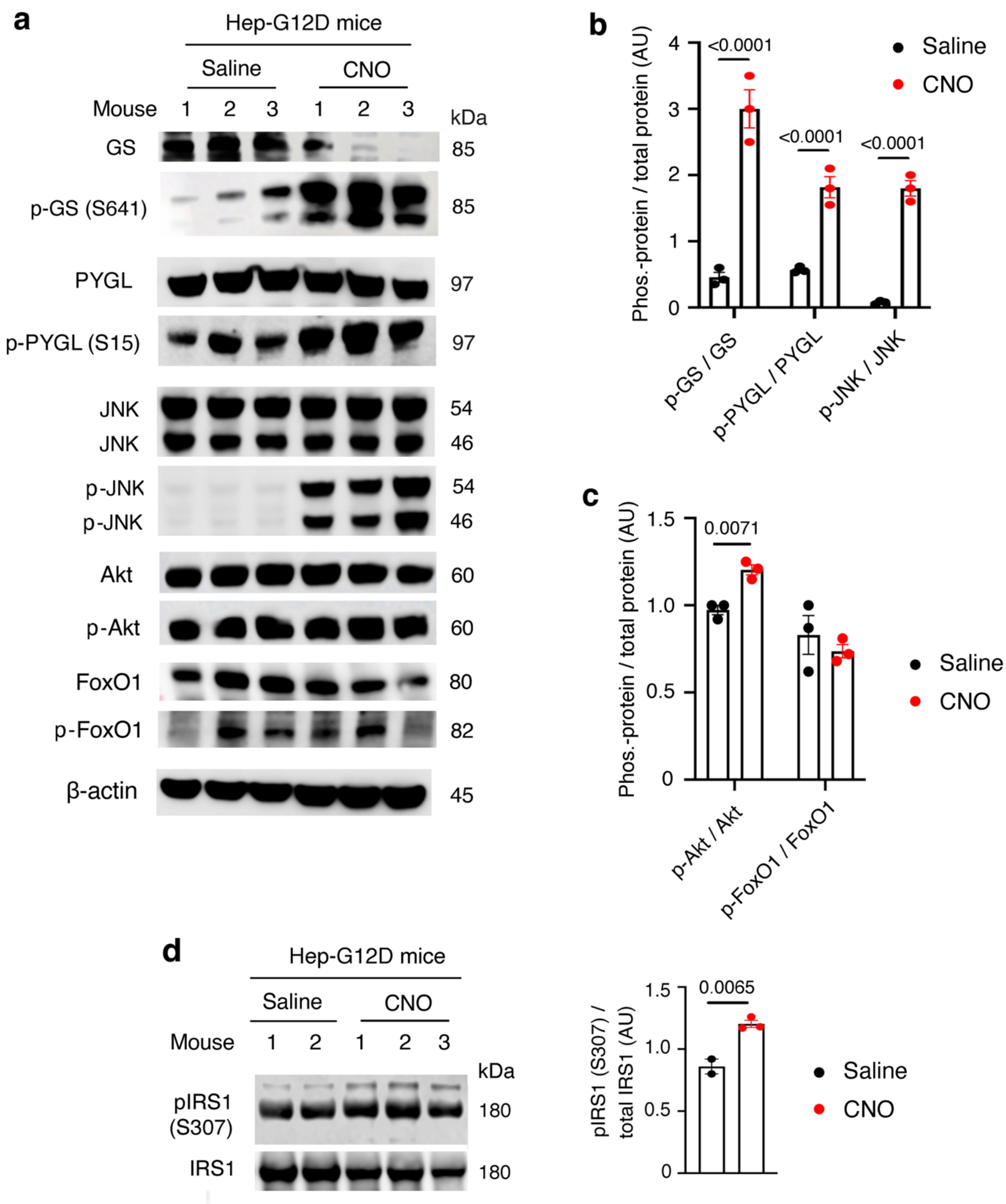

Supplementary Fig. 12. **Activation of G12D expressed by mouse hepatocytes rapidly promotes the phosphorylation of key enzymes and signaling proteins..** HepG12D mice were injected i.v. with either saline or CNO (3 mg/kg). Five min later, mice were euthanized, and liver lysates were prepared and subjected to Western blotting studies. **a, b,** CNO-mediated activation

of G12D led to the rapid phosphorylation of liver glycogen synthase (GS) at position S641 and liver glycogen phosphorylase (PYGL) at position S15, respectively. While pJNK was barely detectable in livers from saline-treated Hep-G12D mice, CNO treatment led to a striking increase in hepatic pJNK formation. **a, c,** CNO-mediated stimulation of G12D had only relatively minor effects on the phosphorylation status of Akt (T308) and FoxO1, two major signaling proteins of the insulin receptor signaling cascade. **d,** CNO treatment of HepG12D mice promoted the phosphorylation of IRS1 at position S307, a target of JNK. Western blotting data are quantified in the bar diagrams. The data shown in the bar graphs represent means ± s.e.m. Numbers above horizontal bars refer to p values (2-way ANOVA followed by Bonferroni's post-hoc test (**b, c**) or two-tailed Student's t test (**d**)). Source data are provided as a Source Data file.

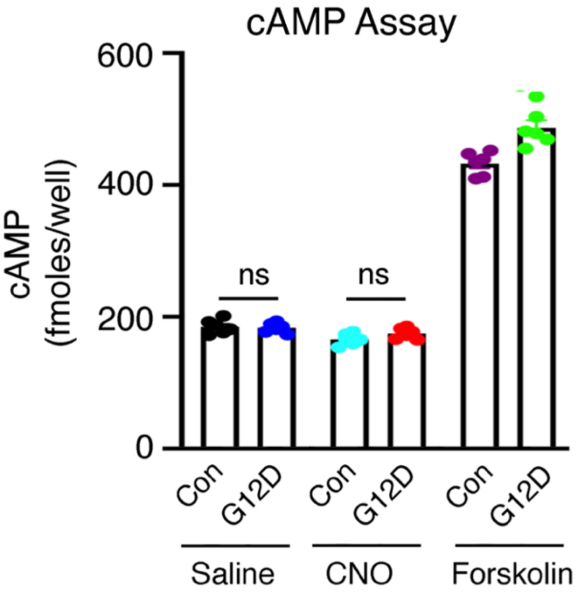

Supplementary Fig. 13. **CNO treatment of Hep-G12D hepatocytes has no effect on intracellular cAMP levels.** CNO treatment (10 µM) of primary hepatocytes prepared from Hep-G12D mice and control littermates (14-week-old males) has no significant effect on intracellular cAMP levels. As expected, incubation with forskolin (10 µM) resulted in a pronounced increase in cytoplasmic cAMP levels (positive control). Cells were incubated with drugs for 30 min. Data are presented as means ± s.e.m. (n=6). ns, no statistically significant difference. Source data are provided as a Source Data file.

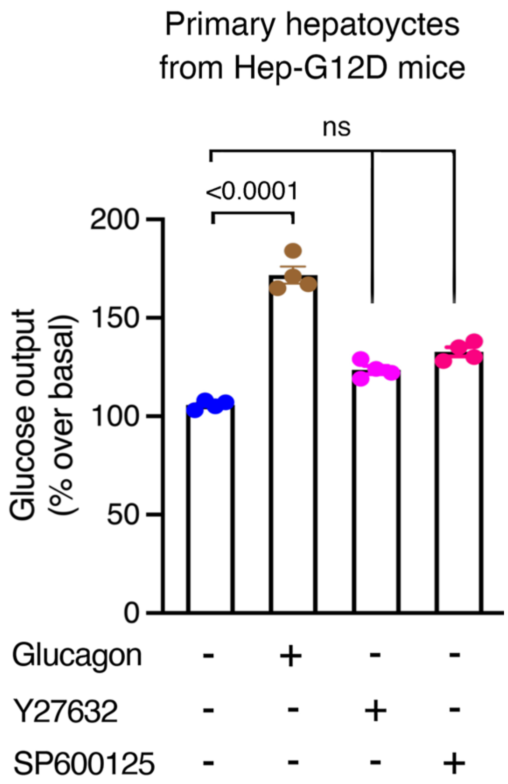

Supplementary 14. Glucose output assays carried out with primary mouse G12D hepatocytes. Primary hepatocytes prepared from Hep-G12D mice were incubated in the presence of glucagon (100 nM; positive control), Y-27632 (10 µM, ROCK inhibitor), or SP600125 (10 µM, JNK inhibitor). Glucose release into the medium was examined 6 hr later (see Methods for details). Note that treatment with Y-27632 or SP600125 alone had no significant effect on glucose output. Data are given means ± s.e.m. from four independent experiments. Numbers above horizontal bars refer to p values (2-way ANOVA followed by Bonferroni's post-hoc test). ns, no statistically significant difference. Source data are provided as a Source Data file.

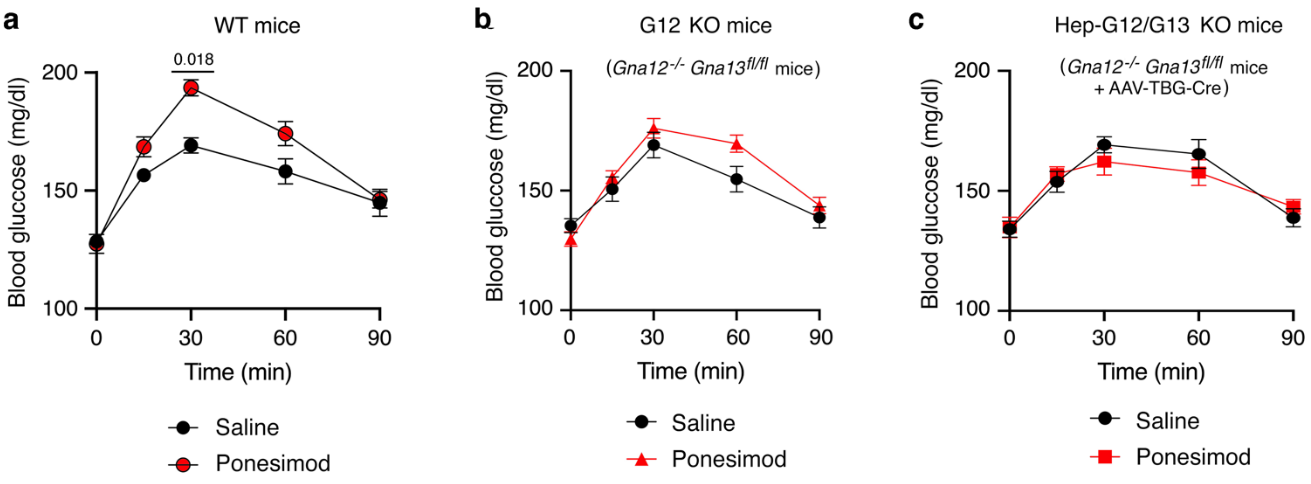

Supplementary Fig. 15. Ponesimod-induced increases in blood glucose levels require $G_{12/13}$ signaling. a-c, Mice (12-14-week-old males) of the indicated genotypes (diet: regular chow) received a single i.p. injection of either saline or ponesimod (20 mg/kg), a selective S1PR1 agonist. Blood glucose levels were determined at the indicated post-injection times.

a, WT mice. **b**, Mice lacking $G\alpha_{12}$ (whole body). **c**, Mice deficient in both $G\alpha_{12}$ (whole body) and $G\alpha_{13}$ (hepatocytes only). Data are given as means ± s.e.m. (n=5 mice/group). Numbers above horizontal bars refer to p values (2-way ANOVA followed by Bonferroni's post-hoc test). Source data are provided as a Source Data file.

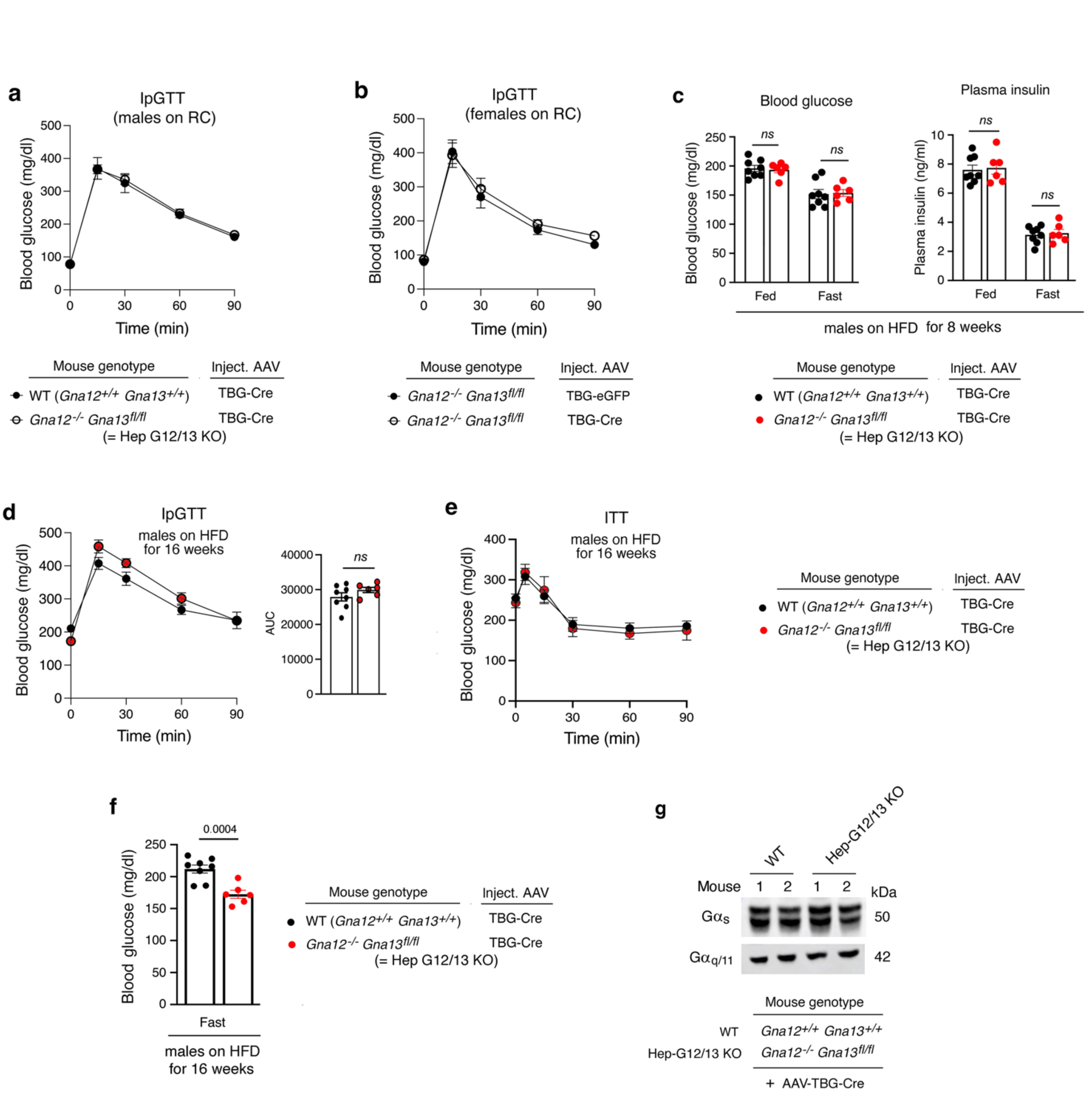

Supplementary Fig. 16. Metabolic studies with mice lacking $G_{12/13}$ signaling in hepatocytes. **a,** IpGTT carried out with male mice of the indicated genotypes following treatment with the AAV-TGG-Cre virus (diet: regular chow [RC]; glucose dose: 2 g/kg). Mice were 12 weeks old when the experiment was performed (n=6-8 per group). **b,** IpGTT performed with female *Gna12*$^{-/-}$ *Gna13*$^{fl/fl}$ mice treated with either the AAV-TGB-eGFP control virus or with AAV-TGG-Cre (diet: RC; glucose dose: 2 g/kg; mouse age: 16 weeks; n=5 per group). **c,** Fed and fasting (14 hr overnight fast) blood glucose and plasma insulin levels of male mice maintained on a high-fat

diet (HFD) for 8 weeks (n=6-8 per group). **d-f,** Metabolic studies performed with male mice maintained on a HFD for 16 weeks (n=6-8 per group). **d,** IpGTT (1 g glucose/kg, i.p.). **e,** ITT (1.5 U Humulin/kg, i.p.). **f,** Fasting blood glucose levels. **g,** Hepatic $G\alpha_s$ and $G\alpha_{q/11}$ protein expression levels remain unaltered in HepG12/13 KO mice (male mice maintained on a HFD for 16 weeks). Data represent means ± s.e.m. Numbers above horizontal bars refer to p values (two-tailed Student's t test). ns, no statistically significant difference. Source data are provided as a Source Data file.

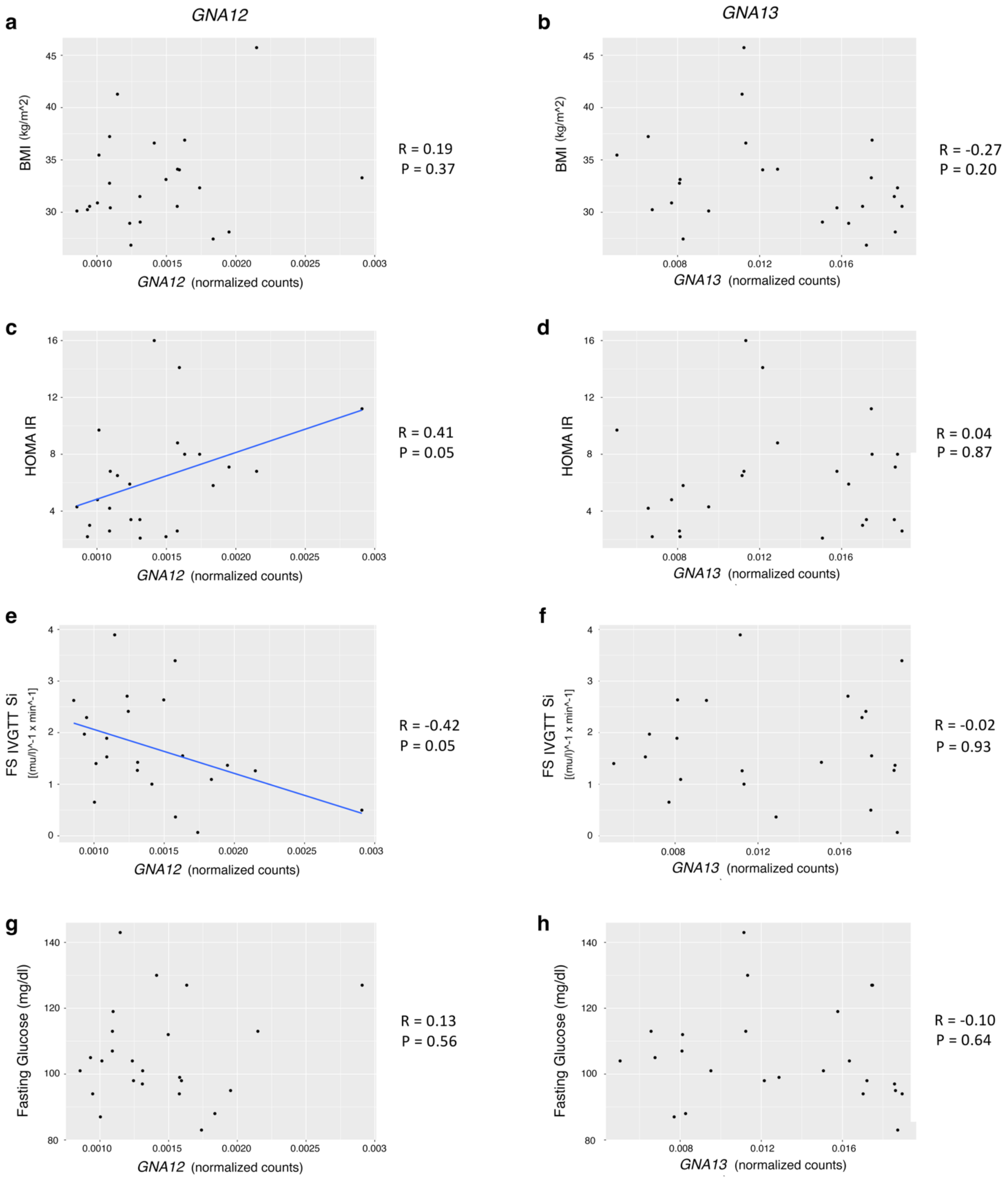

Supplementary Fig. 17. Correlation between hepatic *GNA12* and *GNA13* expression levels and markers of insulin resistance. Liver samples were obtained from human subjects with MASLD differing in glucose tolerance and insulin sensitivity (see Supplementary Table 3). Note that

GNA12 expression levels showed a positive correlation with HOMA-IR and a negative association with FS IVGTT Si. In contrast, *GNA13* expression levels showed no correlation with any of the metabolic parameters analyzed. RNA expression levels were determined via RNAseq. Data were analyzed by Pearson correlations. BMI, body mass index; HOMA-IR, homeostatic model assessment for insulin resistance; FS IVGTT, frequently sampled intravenous glucose tolerance test; MASLD, metabolic dysfunction-associated steatotic liver disease; Si, insulin sensitivity index.

Supplementary Table 1. Summary of antibodies, drugs, reagents, kits, and mouse strains used in this study

Reagent or Resource	Source	Cat #
**Antibodies**		(Dilution in parentheses)
Mouse anti-Gα_{12} antibody (E-12)	Santa Cruz Biotechnology	sc-515445 (1: 2000)
Rabbit anti-Gα_{13} antibody (6F6-B5)	Santa Cruz Biotechnology	sc-540292 (1: 2000)
Anti-mouse IgG, HRP-linked antibody	Cell Signal Technology	7076S (1: 5000)
Anti-Rabbit IgG, HRP-linked antibody	Cell Signal Technology	7074S (1: 5000)
Rabbit anti-ROCK1 antibody	Abcam	Ab134181 (1: 2000)
Rabbit anti-Phospho-SAPK/JNK antibody	Cell Signal Technology	4668S (1: 2000)
Rabbit anti-SAPK/JNK antibody	Cell Signal Technology	9252 (1: 2000)
HA-Tag (C29F4) rabbit mAb	Cell Signal Technology	3724 (1: 2000)
Mouse anti-β-actin antibody	Cell Signaling	3700 (1:5000)
Rabbit anti-HA antibody	Cell Signaling	3724 (1:2000)
HA-Tag (6E2) mouse mAb (Alexa® 488 conjugate)	Cell Signaling	2350s (1:200)
Rabbit anti-phospho-FoxO1 antibody	Cell Signal Technology	9461S
Rabbit anti- FoxO1antibody	Cell Signal Technology	2880S
Rabbit anti-phospho-Akt antibody (T308)	Cell Signal Technology	2965S
Rabbit anti-Akt antibody	Cell Signal Technology	2938S
Rabbit anti-phospho-GSK3β antibody	Cell Signal Technology	9336S
Rabbit anti-GSK3β antibody	Cell Signal Technology	9315S
Rabbit anti-phospho-GS antibody (S641)	Abcam	AB81230
Rabbit anti-GS antibody	Abcam	AB40810
Rabbit anti-phospho-PYGL antibody (S15)	Abcam	AB227043
Rabbit anti-PYGL antibody	Abcam	AB198268
Mouse anti-Gαq antibody	BD Biosciences	612704
Rabbit anti-Gαs antibody	generated in the lab of Dr. Lee S. Weinstein	
Rabbit anti-phospho-IRS-1 antibody (Ser307)	Cell Signaling	2381
Rabbit anti-IRS-1 antibody	Cell Signaling	2382

**Compounds**		
Glucagon	Sigma Aldrich	G2044
Ponesimod	TargetMol	T3258
SP 600125	Tocris Bioscience	1496
Y-27632 dihydrochloride	Tocris Bioscience	1254
Rhosin hydrochloride	Tocris Bioscience	5003
Bovine serum albumin (fatty acid-free)	MilliporeSigma	A7030
Collagenase from *Clostridium histolyticum*	MilliporeSigma	C7657
cOmplete, EDTA-free protease inhibitor cocktail	MilliporeSigma	11697498001
RIPA buffer	Alfa Aesar	AAJ63306AP
EGTA Buffer 0.5 M, pH 8.0	MilliporeSigma	50-255-956
Trypsin 0.25% plus 1 mM EDTA	MilliporeSigma	SM2003C
W146 trifluoroacetate	MedChemExpress LLC	HY-101395A
ECL Western blotting substrate	Thermo Fisher Scientific	32106
PhosSTOP™ (phosphatases inhibitor)	MilliporeSigma	4906845001
Human insulin (Humulin R U-100)	Eli Lilly	NDC 0002-8215-17
Percoll	Sigma Aldrich	P8761
Gibco DMEM, no glucose	Thermo Fisher Scientific	A1443001
Gibco DMEM, low glucose	Thermo Fisher Scientific	11054020
Gibco DMEM, high glucose	Thermo Fisher Scientific	10313039
Tween 20	Fisher Scientific	P7949
TRIzol	Invitrogen	15596026
UK 432097	Axon Medchem	1193
SuperSignal™ West Dura Substrate	Thermo Fisher Scientific	34076
**Kits**		
Ultra-Sensitive Mouse Insulin ELISA kit	Crystal Chem	90082
Glucose (HK) assay kit	Sigma Aldrich	GAHK20
ON-TARGETplus Mouse Gna12 (14673) siRNA - SMARTpool	Horizon Discovery	L-043467-00-0005
ON-TARGETplus Non-targeting Pool	Horizon Discovery	D-001810-10-05
Glycogen phosphorylase colorimetric assay kit	BioVision	K179
GSK3ß assay kit	BPS Bioscience	79700

ROCK activity assay kit	Sigma Aldrich	CSA001
BCA protein assay kit	Thermo Fisher Scientific	23225
Glucose 6 phosphate assay kit, colorimetric	Novus Biologicals	NBP3-24492
cAMP, Biotrak™ EIA System	GE HealthCcare	RPN225
**Experimental Models: Mouse Strains**		
C57BL/6N mice (WT mice)	Taconic	C57BL/6NTac
ROSA26-LSL-G12D-IRES-GFP mice	Source: Dr. Asuka Inoue	Ref[1]
Jnk1/2 fl/fl mice	Source: Dr. Roger Davis	Ref[4]
Rock1 fl/fl mice	Source: Dr. Young-Bum Kim	Ref[5]
Gna12-/- Gna13 fl/fl mice	from Dr. Stefan Offermanns	Ref[6]
Gna13 fl/fl mice	generated at NIDDK in the Wess section from *Gna12-/- Gna13 fl/fl* mice	
**Adeno-associated viruses and adenoviruses**		
AAV.TBG.PI.Cre.rBG	Addgene	107787-AAV8
AAV.TBG.PI.eGFP.WPRE.bGH	Addgene	105535-AAV8
Ad-CMV-eGFP	Vector Biolabs	Custom order
Ad-CMV-HA-G12D	Vector Biolabs	Custom order

Supplementary Table 2. Primers and TaqMan reagents used for PCR/qRT-PCR experiments

**Gene**	**Forward primer (5'-3')**	**Reverse primer (5'-3')**
G6pc	AGGTCGTGGCTGGAGTCTTGTC	GTAGCAGGTAGAATCCAAGCGC
Fbp1	TGCTGAAGTCGTCCTACGCTAC	TTCCGATGGACACAAGGCAGTC
Pck	GGCGATGACATTGCCTGGATGA	TGTCTTCACTGAGGTGCCAGGA
G12D	GTACCACCGATGACCCTCTG	CCAGGATGTTGCCGATGATG
Gcgr	CAATGCCACCACAACCTAAGCC	GGCAGGAAATGTTGGCAGTGGT
Avrp1a	CATCCTCTGCTGGACACCTTTC	TCAAGGAAGCCAGTAACGCCGT
S1p1r	CGCAGTTCTGAGAAGTCTCTGG	GGATGTCACAGGTCTTCGCCTT
Gpr91	CCATCTCTGACTTTGCTTTCCTG	GTGTAGAGGTTGGTGTGAAGCAC

**Gene symbol**	**Encoded protein**	**TaqMan Assay ID**
Gna12	$G\alpha_{12}$	Hs04992528_s1
Gna13	$G\alpha_{13}$	Hs00183573_m1
Rock1	Rho associated coiled-coil containing protein kinase 1	Hs01127701_m1
Rock2	Rho associated coiled-coil containing protein kinase 2	Hs00178154_m1
RhoA	Ras homolog family member A (RhoA)	Hs00357608_m1
Jnk1	c-Jun N-terminal kinase 1	Ce02407570_m1

Supplementary Table 3. Characteristics of human subjects from which liver biopsy samples were obtained

Age [years]	49±12
Male Sex [n, %]	14 (58%)
Race/Ethnicity	
White	8 (33%)
Hispanic	15 (63%)
Asian	1 (4%)
BMI [kg/m^2]	32.8±4.4
Hemoglobin A1c [%]	5.7±0.4
HOMA-IR	6.3±3.7
Si Insulin Sensitivity Index	1.7±1
Histological Steatosis Score (median, range)[1]	1 (0-3)
NAFLD Activity Score (NAS) (median, range)[a]	3 (1-6)

[a]Scored according to Kleiner et al.[7].

Data are given as means± s.d. (n=24). Abbreviations used: BMI, body mass index; HOMA-IR, homeostatic model assessment for insulin resistance; NAFLD, non-alcoholic fatty liver disease.

References

1. Ono, Y., *et al.* Chemogenetic activation of G(12) signaling enhances adipose tissue browning. *Signal Transduct Target Ther* **8**, 307 (2023).
2. Inoue, A., *et al.* Illuminating G-protein-coupling selectivity of GPCRs. *Cell* **177**, 1933-1947.e1925 (2019).
3. Armbruster, B.N., Li, X., Pausch, M.H., Herlitze, S. & Roth, B.L. Evolving the lock to fit the key to create a family of G protein-coupled receptors potently activated by an inert ligand. *Proc Natl Acad Sci U S A* **104**, 5163-5168 (2007).
4. Han, M.S., *et al.* JNK Expression by macrophages promotes obesity-induced insulin resistance and inflammation. *Science* **339**, 218-222 (2013).

5. Huang, H., *et al.* Rho-kinase regulates energy balance by targeting hypothalamic leptin receptor signaling. *Nat Neurosci* **15**, 1391-1398 (2012).
6. Wirth, A., *et al.* G12-G13-LARG-mediated signaling in vascular smooth muscle is required for salt-induced hypertension. *Nat Med* **14**, 64-68 (2008).
7. Kleiner, D.E., *et al.* Design and validation of a histological scoring system for nonalcoholic fatty liver disease. *Hepatology* **41**, 1313-1321 (2005).
