# Supplementary material for: G12/13-mediated signaling stimulates hepatic glucose production and has a major impact on whole body glucose homeostasis
Source: Nat Commun. 2024 Nov 19;15:9996. doi: 10.1038/s41467-024-54299-7 (PMC11574106; doi:10.1038/s41467-024-54299-7)
Supplement: Supplementary file 2 — Reporting Summary [file 41467_2024_54299_MOESM2_ESM.pdf]

nature portfolio

Corresponding author(s): Jürgen Wess

Last updated by author(s): 11/05/2024

Reporting Summary

Nature Portfolio wishes to improve the reproducibility of the work that we publish. This form provides structure for consistency and transparency in reporting. For further information on Nature Portfolio policies, see our Editorial Policies and the Editorial Policy Checklist.

Statistics

For all statistical analyses, confirm that the following items are present in the figure legend, table legend, main text, or Methods section.

n/a	Confirmed	
☐	☒	The exact sample size (*n*) for each experimental group/condition, given as a discrete number and unit of measurement
☐	☒	A statement on whether measurements were taken from distinct samples or whether the same sample was measured repeatedly
☐	☒	The statistical test(s) used AND whether they are one- or two-sided<br>*Only common tests should be described solely by name; describe more complex techniques in the Methods section.*
☐	☒	A description of all covariates tested
☐	☒	A description of any assumptions or corrections, such as tests of normality and adjustment for multiple comparisons
☐	☒	A full description of the statistical parameters including central tendency (e.g. means) or other basic estimates (e.g. regression coefficient) AND variation (e.g. standard deviation) or associated estimates of uncertainty (e.g. confidence intervals)
☐	☒	For null hypothesis testing, the test statistic (e.g. *F*, *t*, *r*) with confidence intervals, effect sizes, degrees of freedom and *P* value noted<br>*Give P values as exact values whenever suitable.*
☒	☐	For Bayesian analysis, information on the choice of priors and Markov chain Monte Carlo settings
☒	☐	For hierarchical and complex designs, identification of the appropriate level for tests and full reporting of outcomes
☐	☒	Estimates of effect sizes (e.g. Cohen's *d*, Pearson's *r*), indicating how they were calculated

Our web collection on statistics for biologists contains articles on many of the points above.

Software and code

Policy information about availability of computer code

Data collection	Prism9, Excel
Data analysis	Prism9, Excel, ImageJ

For manuscripts utilizing custom algorithms or software that are central to the research but not yet described in published literature, software must be made available to editors and reviewers. We strongly encourage code deposition in a community repository (e.g. GitHub). See the Nature Portfolio guidelines for submitting code & software for further information.

Data

Policy information about availability of data

All manuscripts must include a data availability statement. This statement should provide the following information, where applicable:

- Accession codes, unique identifiers, or web links for publicly available datasets
- A description of any restrictions on data availability
- For clinical datasets or third party data, please ensure that the statement adheres to our policy

Source data for all figures will be provided with the paper. All other data supporting the findings of this study are available from the authors upon request.

Research involving human participants, their data, or biological material

Policy information about studies with human participants or human data. See also policy information about sex, gender (identity/presentation), and sexual orientation and race, ethnicity and racism.

Reporting on sex and gender	Sex of human subjects is reported in Supplementary Table 3. The sample size does not allow sufficient power for sex- or gender-based analyses.
Reporting on race, ethnicity, or other socially relevant groupings	Race and ethnicity of human subjects is reported in Supplementary Table 3 based on self-reported identity.
Population characteristics	Reported in Supplementary Table 3
Recruitment	All study participants for whom there was sufficient liver RNA for RNA-sequencing were included in the analysis
Ethics oversight	The study was approved by the NIDDK/NIAMS Institutional Review Board, and all subjects provided written informed consent.

Note that full information on the approval of the study protocol must also be provided in the manuscript.

Field-specific reporting

Please select the one below that is the best fit for your research. If you are not sure, read the appropriate sections before making your selection.

☒ Life sciences ☐ Behavioural & social sciences ☐ Ecological, evolutionary & environmental sciences

For a reference copy of the document with all sections, see nature.com/documents/nr-reporting-summary-flat.pdf

Life sciences study design

All studies must disclose on these points even when the disclosure is negative.

Sample size	Sample size was chosen based on prior experience of the investigators with similar experiments previously published. The authors have published numerous peer-reviewed papers demonstrating clear positive findings with similar sample sizes for the types of experiments included.
Data exclusions	No data points were excluded from the analysis of any of the experiments.
Replication	All experimental findings were reproduced in several independent experiments, as indicated in the figure legends.
Randomization	Randomization was performed by blinding investigators to genotype and allowing them to choose each subject blindly.
Blinding	Randomization was performed by blinding investigators to genotype and allowing them to choose each subject blindly. For studies using pharmacological agents, the investigator was aware of the agent being used, but was not aware of the genotypes of the animals used. Investigators were not aware of the specific group to which an animal was assigned to when doing the experiment or until after completion of the experiment.

Reporting for specific materials, systems and methods

We require information from authors about some types of materials, experimental systems and methods used in many studies. Here, indicate whether each material, system or method listed is relevant to your study. If you are not sure if a list item applies to your research, read the appropriate section before selecting a response.

Materials & experimental systems

n/a	Involved in the study	
☐	☒	Antibodies
☐	☒	Eukaryotic cell lines
☒	☐	Palaeontology and archaeology
☐	☒	Animals and other organisms
☐	☒	Clinical data
☒	☐	Dual use research of concern
☒	☐	Plants

Methods

n/a	Involved in the study	
☒	☐	ChIP-seq
☒	☐	Flow cytometry
☒	☐	MRI-based neuroimaging

Antibodies

Antibodies used	The following primary antibodies were used:

Mouse anti-Galpha-12 antibody (E-12) - Santa Cruz Biotechnology sc-515445 (1: 2000)
Rabbit anti-Galpha-13 antibody (6F6-B5) - Santa Cruz Biotechnology sc-540292 (1: 2000)
Rabbit anti-ROCK1 antibody - Abcam Ab134181 (1: 2000)
Rabbit anti-Phospho-SAPK/JNK antibody - Cell Signal Technology 4668S (1: 2000)
Rabbit anti-SAPK/JNK antibody - Cell Signal Technology 9252 (1: 2000)
Rabbit Anti-HA-Tag (C29F4) mAb - Cell Signal Technology 3724 (1: 2000)
Mouse anti-β-actin antibody - Cell Signaling 3700 (1:5000)
Rabbit anti-HA antibody - Cell Signaling 3724 (1:2000)
HA-Tag (6E2) mouse mAb (Alexa® 488 conjugate) - Cell Signaling 2350s (1:200)
Rabbit anti-phospho-FoxO1 antibody - Cell Signal Technology 9461S
Rabbit anti- FoxO1antibody - Cell Signal Technology 2880S
Rabbit anti-phospho-Akt antibody (T308) - Cell Signal Technology 2965S
Rabbit anti-Akt antibody - Cell Signal Technology 2938S
Rabbit anti-phospho-GSK3beta antibody - Cell Signal Technology 9336S
Rabbit anti-GSK3beta antibody - Cell Signal Technology 9315S
Rabbit anti-phospho-glycogen synthase antibody (S641) - Abcam AB81230
Rabbit glycogen synthase antibody - Abcam AB40810
Rabbit anti-phospho-PYGL antibody (S15) - Abcam AB227043
Rabbit anti-PYGL antibody Abcam AB198268
Mouse anti-Galpha-q antibody - BD Biosciences 612704
Rabbit anti- Galpha-s antibody - generated in the lab of Dr. Lee S. Weinstein
Rabbit anti-phospho-IRS-1 antibody (Ser307) - Cell Signaling 2381
Rabbit anti-IRS-1 antibody - Cell Signaling 2382

Validation	Validated by the manufacturer (for details, please visit the manufacturers' web sites)

Eukaryotic cell lines

Policy information about cell lines and Sex and Gender in Research

Cell line source(s)	HepG2 cells (ATCC, cat # HB-8065)
Authentication	authenticated by ATCC
Mycoplasma contamination	The cell line tested negative for mycoplasma contamination.
Commonly misidentified lines (See ICLAC register)	No commonly misidentified cell lines were used in the study.

Animals and other research organisms

Policy information about studies involving animals; ARRIVE guidelines recommended for reporting animal research, and Sex and Gender in Research

Laboratory animals	Male and female mice (C57BL6 background) older than 8 weeks; see the Methods section for detailed housing conditions
Wild animals	No wild animals were used in the study.
Reporting on sex	Key experiments were carried out with both male and female mice. Data for male and female mice were analyzed separately. Similar results were obtained for male and female mice. Sex was determined by anatomical analysis. Key data for female mice are shown in Supplementary Fig. 6.
Field-collected samples	No field collected samples were used in the study.
Ethics oversight	All animal studies were approved by the NIDDK institutional Animal Care and Use Committee.

Note that full information on the approval of the study protocol must also be provided in the manuscript.

Clinical data

Policy information about clinical studies
All manuscripts should comply with the ICMJEguidelines for publication of clinical research and a completedCONSORT checklist must be included with all submissions.

Clinical trial registration	NCT01792115
Study protocol	Protocol available from author YR
Data collection	Data collected at NIH Clinical Center 2013-2017

Outcomes	N/A - this is a secondary analysis

Plants

Seed stocks	N/A
Novel plant genotypes	N/A
Authentication	N/A
